# Death-related gastric necrosis after Laparoscopic Adjustable Gastric Banding in the early post-operative period

**DOI:** 10.1186/1746-1596-5-68

**Published:** 2010-10-11

**Authors:** Kleio Fragkouli, Antigoni Mitselou, Theodore Vougiouklakis

**Affiliations:** 1 Department of Forensic Medicine and Toxicology, Medical School, University of Ioannina, 451 10 Ioannina, Greece

## Abstract

We report a case of a rare complication of Laparoscopic Adjustable Gastric Banding (LAGB) as a cause of death in the immediate post-operative period. The number of relevant reports and postmortem images presented in the literature is extremely restricted. Gastric necrosis may constitute a cause of death after LAGB in the early post-operative period. Postmortem examination reveals the extension of gastric ischemia and necrosis, responsible for the lethal outcome. To date, only one case of gastric necrosis after LAGB in the immediate post-operative period leading to death has been reported, according to authors' knowledge. The diagnosis of this complication may be delayed on the grounds of its rarity. In our opinion, surgeons should be aware of the clinical state implying gastric ischemia early after LAGB, so as to recognize and, in turn, to treat it promptly.

## Short Report

A considerably high number of surgeons worldwide prefer to treat morbid obesity by means of Laparoscopic Adjustable Gastric Banding (LAGB), a restrictive bariatric technique used to reduce gastric volume. The procedure's technical simplicity and relative safety have rendered it one of the most common surgical techniques in Europe, Australia, Latin America and, recently, in USA [1-3]. However, despite its popularity, LAGB is related to serious complications, both early and late, that usually raise the demand of a second operation or even lead to a fatal outcome if not recognized [4]. Latest data place the incidence of LAGB's complications at the figure of 12.2% [5]. The most frequent complications are device-related and, in recent years, their incidence appears essentially decreased, owing to modifications in the surgical approach. Band slippage/gastric prolapse presents the highest reported frequency of approximately 4.5 - 5% [5]. Among other complications, bleeding, pouch dilatation, anastomotic leaks and gastric perforation are included. On the other hand, although data about mortality after LAGB is rather restricted, the recently reported percentages range from 0.34% to 0.51% and the most common causes of death are pulmonary embolism and myocardial infarction [4,5].

Gastric necrosis represents a rare but life-threatening complication of gastric banding, that may appear in the early post-operative period and it is possible to lead to death if untreated.

**Figure 1:**
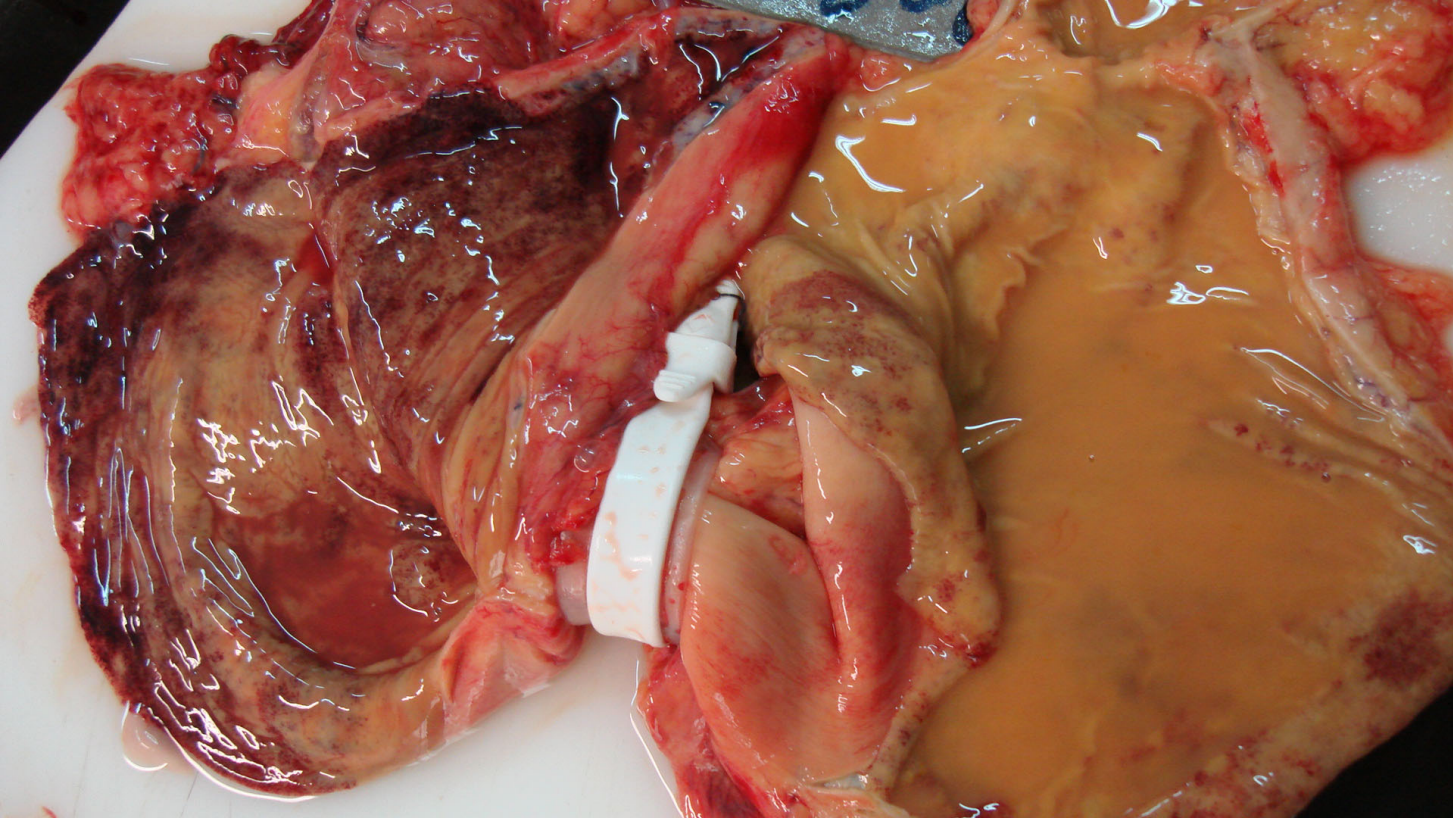
**Photograph showing the macroscopic aspect of gastric mucosa proximally and distally to the band, after the incision of the stomach**.

**Figure 2:**
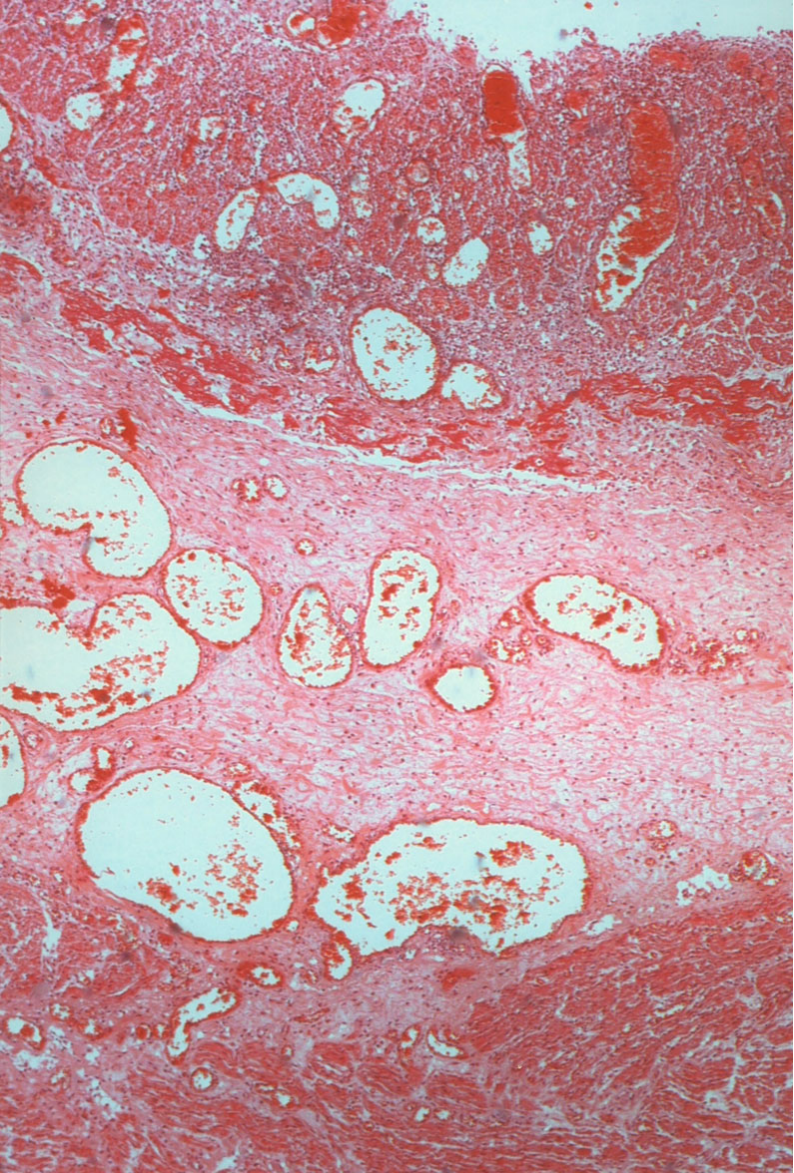
**Histological appearance of gastric wall proximally to the band, showing mucosal necrosis, extensive submucosal edema, dilated vessels, inflammatory infiltrate and edema of the muscularis layer (Hematoxylin-Eosin stain, original magnification ×40)**.

A 37-year-old man presented to ED reporting a 2 days' vomiting, abdominal pain and discomfort that had progressively worsened. He had undergone LABG for morbid obesity 8 days previously. His body temperature was 39°C, blood pressure 80/50 mmHg and he had 110 beats per minute. On physical examination signs of peritoneal irritation were observed. Laboratory findings included a white blood cell count of 15,000 μ/L and an LDH value of 590. His clinical state deteriorated rapidly and despite the urgent surgery, he died during surgical exploration, as a consequence of respiratory insufficiency. His body was transferred to our Department for post-mortem examination. Autopsy revealed a gastric band placed around the middle part of the gastric body (Figure 1). The mucosa at the level of cardio-esophageal junction showed patchy hemorrhages, whereas anterior and posterior gastric wall proximally to the band appeared excessively hemorrhagic with numerous erosions but without macroscopic signs of perforation. The gastric mucosa distally to the band presented loss of its natural plication along with scattered hemorrhages. Other autopsy findings included signs of septicemia. The principal histological findings were ischemic necrosis of the mucosa of the lower third of esophagus (including the cardia) and mucosal necrosis and severe ischemic lesions of the remaining layers of the gastric wall proximally to the band (Figure 2). The part of gastric mucosa distally to the band revealed mild edema and microscopic features of chronic gastritis. Toxicological analysis was negative.

Gastric necrosis is a rare complication of LAGB; its diagnosis is often delayed and, when present, comprises a medical emergency. Although the rich blood supply of the stomach, with its extensive intramural anastomoses, render the organ resistant to ischemia, a combination of comorbidities existing in obese individuals along with the risks of LAGB itself may result in gastric necrosis.

To our knowledge, only one case of gastric necrosis after LABG in the immediate post-operative period leading to death has been reported to date [6].

Conclusively, gastric necrosis after LAGB constitutes a potentially lethal complication of the procedure, emerging even as a short-term one. In our opinion, surgeons should be aware of any clinical signs indicating a gastric necrosis so as to recognize it promptly and manage it properly.

## Competing interests

The authors declare that they have no competing interests.

## Authors' contributions

KF constructed the majority of the manuscript, and edited the entire draft for final submission. AM performed the microscopic analysis, and acquired photomicrographs. TV was the forensic pathologist responsible for the autopsy case and was responsible for a significant portion of the manuscript. All authors read and approved the final manuscript.
